# A simple HPLC–MS/MS method for the determination of polymyxin B in human plasma and its application in the pharmacokinetic study in elderly patients infected with multidrug-resistant Gram-negative bacteria

**DOI:** 10.3389/fphar.2024.1396307

**Published:** 2024-08-16

**Authors:** Sheng Hu, Nan Guo, Juan Zeng, Yue Li, Yahui Zhang, Jinjiao Jiang, Bing Leng, Chengwu Shen

**Affiliations:** ^1^ Department of Pharmacy, Shandong Provincial Hospital Affiliated to Shandong First Medical University, Jinan, China; ^2^ Department of Graduate, Shandong First Medical University and Shandong Academy of Medical Sciences, Jinan, China; ^3^ Department of Critical Care Medicine, Shandong Provincial Hospital Affiliated to Shandong First Medical University, Jinan, China

**Keywords:** polymyxin B, HPLC–MS/MS, pharmacokinetics, elderly patients, dose

## Abstract

**Introduction:** Polymyxin B is widely used to treat infections caused by multidrug-resistant Gram-negative bacteria. However, the pharmacokinetic study data of PB in the elderly are scarce. Herein, a simple method to measure the concentration of PB in human plasma was developed and validated by high performance liquid chromatography—tandem mass spectrometry, and it was applied to a PK study in the elderly.

**Methods:** PB was extracted from human plasma by a rapid protein-precipitation method using 0.1% formic acid in methanol and then separated on an ultimate AQ-C18 column using linear gradient elution with a 0.5-mL/min flow rate. Subsequently, PB was detected using a mass spectrometer operated in positive-ion and multiple-reaction-monitoring modes.

**Results:** The lower limits of quantification of the method for Polymyxin B1 and Polymyxin B2 were 1.00 and 0.10 μg/mL, respectively. The linear ranges for PB1 and PB2 were 1.00–20.02 and 0.10–2.04 μg/mL, respectively. Patients receiving a 75-mg maintenance dose every 12h had AUC_ss_, 24 h, and C_ss, av_ values of 117.70 ± 37.03 μg h/mL and 4.14 ± 1.74 μg/mL, respectively. For patients receiving a 100 mg maintenance dose, these values were 152.73 ± 70.09 μg h/mL and 5.43 ± 2.85 μg/mL, respectively.

**Conclusion:** The validated HPLC–MS/MS method was successfully applied to a study on the pharmacokinetics of PB in elderly patients infected with multidrug-resistant Gram-negative bacteria. Both two dose strategies in this study would have a excessive PB exposure in the elderly patients then the therapeutic window recommended by guidelines.

## 1 Introduction

Polymyxin B (PB), a cyclic cationic polypeptide whose major components are polymyxin B1 (PB1), polymyxin B2 (PB2), polymyxin B3 (PB3), and polymyxin B1-ile (PB1-ile), was used as a last-line treatment for infections caused by multidrug-resistant Gram-negative bacteria, including *Klebsiella pneumoniae*, *Pseudomonas aeruginosa*, and *Acinetobacter baumannii* (Li et al., 2006). It was abandoned by clinicians in the 1970s due to its narrow therapeutic window and serious renal adverse effects (Falagas and Kasiakou, 2006). In recent years, multidrug-resistant bacteria have emerged because of the use and misuse of antibiotics. Despite the recent approval of new antibacterial agents, there is still an urgent need for therapeutic options to manage antimicrobial threat, which has led to an increasing use of older antibiotics such as PB. The optimal dose of PB remains to be refined and improved. Therefore, robust pharmacokinetic (PK) knowledge and practical therapeutic drug monitoring (TDM) methods are necessary to optimize the clinical use of PB (Tsuji et al., 2019).

High-performance liquid chromatography–tandem mass spectrometry (HPLC–MS/MS) has been used for determining PB concentrations in clinical samples (Hee et al., 2017; Liu et al., 2020; Zhang et al., 2023). Hee et al. (2017) used a Zorbax Bonus-RP C14 column to enhance the separation and quantification of PB1, PB2, PB3, and PB1-ile. However, the column used in their method is expensive. Moreover, trichloroacetic acid (TCA) was used as a protein precipitant to enhance the recovery of PBs, but TCA deposition in a mass spectrometer may damage the instrument. Liu et al. (2020) used solid-phase extraction as a sample pretreatment method, which is costly and complex for a large number of clinical samples. Wang et al. (2020a) developed an analytical method using protein precipitation as a simple and rapid sample-pretreatment method. However, they used a single PB component as the standard reference, which is more expensive and less easily available on the market than the mixture of PB. This study aims to develop a simple, rapid, and economical method for the routine TDM of PB.

Elderly individuals are susceptible to infectious diseases, particularly those caused by multidrug-resistant bacteria, due to age-related tissue-and-organ degeneration and a decline in immune function (Yoshikawa and Norman, 2017). Owing to the age-related physiological changes in elderly patients (including reduced hepatic and renal function, hypoproteinemia, and hemodynamic instability), the PK of antibiotics in this population may differ from those in adults (Pea, 2018). Currently, there are a limited number of studies on the pharmacokinetics of PB in elderly patients. Hence, this study used the validated method to investigate PB pharmacokinetics in elderly patients infected with multidrug-resistant Gram-negative bacteria.

## 2 Materials and methods

### 2.1 Reagents and chemicals

Polymyxin B sulfate (Lot: R104F0) was purchased from the United States Pharmacopeia. The composition of the 1-mg PB standard was as follows: 0.741 mg of PB1, 0.083 mg of PB2, and 0.073 mg of PB1-ile. Carbamazepine (lot: 100142–201706, purity: 100%) was obtained from the Chinese National Institutes for Food and Drug Control (China), and it was used as an internal standard (IS). Ultrapure water was acquired from Merck Millipore (United States). HPLC-grade methanol was provided by Merck (Germany). HPLC-grade formic acid (FA) was supplied by Fisher Scientific (Belgium).

### 2.2 HPLC–MS/MS conditions

An Agilent 1260 Infinity II liquid chromatography system and an Agilent 6460 triple quad mass spectrometer were used for HPLC–MS/MS analysis. Chromatographic separation was performed under gradient elution with a 0.5-mL/min flow rate, and the column oven temperature was maintained at 30°C. PB1, PB2, and the IS were separated by an Ultimate AQ-C18 column (3.0 mm × 100 mm, 3 μm; Welch). Mobile phase A consisted of 0.1% FA in water, and mobile phase B consisted of 0.1% FA in methanol. The MS conditions were set as follows: nitrogen drying gas temperature: 350°C; nitrogen drying gas flow: 11 mL/min; fragmentor: 150 eV for PB1, 135 eV for PB2, and 135 eV for IS; collision energy (CE): 19 eV for PB1, 22 eV for PB2, and 15 eV for IS; dwell time: 100 ms. Transitions ion pair m/z 602.3 → 101.0 for PB1, m/z 595.5 → 101.0 for PB2, and m/z 236.5 → 194.0 for IS were performed in the positive ESI and MRM modes. The gradient elution program was conducted as follows: 0–0.5 min, 70%–5% A, 0.5–3.0 min, 5% A, 3.0–3.5 min, 5%–70% B, and held at 70% A until the end of the run. Under this method, the typical retention time durations for PB1 and PB2 were 3.1 min and 3.8 min for IS, respectively. The injection volume was 10.0 μL, and the run time for each injection was 4.2 min, with 3.3 min post-run time. The total run time for each injection was 7.5 min.

### 2.3 Calibration standards and quality control samples

The stock solutions of the calibrators and quality control (QC) samples should be prepared separately in the same way. The PB stock solution for calibrators and QC samples was prepared in water (1% FA) at 400.48 μg/mL for PB1 and 40.84 μg/mL for PB2. Consequently, working solutions for construction of calibrators and QC were prepared by serially diluting the stock solution with a 1% aqueous solution of FA. The concentrations of the working solutions for calibrators were 10.01, 30.03, 50.05, 100.12, 150.18, and 200.24 μg/mL for PB1 and 1.02, 3.06, 5.10, 10.21, 15.31, and 20.42 μg/mL for PB2. The concentrations of working solutions for QCs were prepared at 20.02 μg/mL (LQC), 90.11 μg/mL (MQC), and 180.22 μg/mL (HQC) for PB1 and 2.04 μg/mL (LQC), 9.18 μg/mL (MQC), and 18.36 μg/mL (HQC) for PB2. The working solution of IS (10.00 μg/mL) was prepared using methanol. All the above solutions were stored at −70°C. The calibrators and QC samples were obtained by spiking 90 μL of blank plasma with 10 μL of working solution at the corresponding concentration. The standard curves were in the range of 1.00–20.02 μg/mL and 0.10–2.04 μg/mL for PB1 and PB2, respectively.

### 2.4 Sample preparation

Typically, 100 μL of the plasma sample was mixed with 10 μL IS working solution, and then, 300 μL 0.1% FA in methanol was added to the sample for protein precipitation. The mixture was vortexed for 3 min and centrifuged in an Eppendorf 5415D microcentrifuge (Eppendorf, Hamburg, Germany) at 11,000 g rpm for 10 min. Subsequently, 200 μL of the supernatant was transferred into a clean polypropylene tube and diluted with 150 μL of 0.1% FA in water. The mixture solution was vortexed for 3 min and centrifuged at 11,000 rpm for 10 min using the same centrifuge. Finally, 200 μL of the supernatant was transferred into an injection bottle, and 10 μL was injected into the HPLC–MS/MS for analysis.

### 2.5 Method validation

The method validation was performed according to the Chinese Pharmacopeia 9012 Guidelines for Validation of Quantitative Analytical Method of Biological Samples and ICH M10 Guidelines on Bioanalytical Method Validation (YDZ, 2020; European Medicines Agency, 2022). The validation included the following parameters: specificity, selectivity, linearity, accuracy, precision, matrix effects, recovery, carryover, dilution integrity, and stability. To investigate the potential endogenous interference from co-administered drugs in the plasma, blank plasma spiked with five antibiotics (tigecycline, meropenem, cefoperazone, voriconazole, and linezolid), samples spiked with two analytes and IS, and samples obtained after drug administration were compared based on the retention time responses of two analytes and IS. The selectivity was determined by analyzing the blank plasma from six different sources, checking for interfering signals at the retention times of the analyte and IS. Potential endogenous interferences were evaluated by analyzing the lower limit of quantification (LLOQ) sample with IS and the blank sample spiked with only IS, respectively. The interference peak area in the blank samples should be lower than 20% of the analyte peak at LLOQ and 5% for IS. Linearity was estimated by assaying standard calibration samples at six non-zero concentration levels for three consecutive days, the mass spectral peak area of the analyte was taken as the ordinate, and a linear regression standard curve was drawn with the concentration of the analyte as the abscissa, from which the regression equation was obtained. The LLOQ was accepted as the concentration of the lowest calibration standard if the analyte response was identifiable, discrete, and reproducible and the signal-to-noise ratios for all LLOQ samples were greater than 10. Intra- and inter-day accuracy and precision were assessed by evaluating six replicate QC samples at LLOQ, LQC, MQC, and HQC concentrations within three successive days. The accuracy was measured by relative error (RE), which should be within ±15% of the QC samples and ± 20% for LLOQ. The precision was evaluated by relative standard deviation (RSD), which should not exceed 15%.

The recoveries of PB1 or PB2 were determined by comparing the responses of processed QC samples at three levels (LQC, MQC, and HQC) with those in post-extracted plasma spiked with working solutions at the corresponding concentration. The recovery of the IS was calculated similarly. The matrix effect was calculated by comparing the responses of analytes added to post-extracted blank plasma replicates from six different resources with those in neat solutions at three QC concentrations. The matrix effects of hyperlipemic plasma and hemolysed plasma samples were also evaluated in this study. Hyperlipemic matrix were obtained from the Shandong Institute for Food and Drug Control. Hemolysed matrix was obtained by spiking a blank plasma sample with hemolysed whole blood (2% v/v). The matrix factor of the IS was calculated similarly. The IS-normalized matrix factors were calculated by dividing the matrix factors of the analyte by that of the IS.

Carryover was assessed by injecting a blank sample after three consecutive injections of the ULOQ samples. The response of the blank sample should be less than 20% of the LLOQ of the analyte and less than 5% of the IS. No significant carryover was observed throughout the validation process.

To assess dilution integrity, plasma samples were prepared by adding 36.04 μg/mL of PB1 and 3.68 μg/mL of PB2 (concentrations 2-fold higher than HQC) to blank plasma. Subsequently, these plasma samples were diluted to the HQC level using blank plasma.

The stability of the working solutions of PB1 and PB2 was evaluated. The short-term stability and long-term stability of PB1 and PB2 in human plasma were evaluated under the following conditions: placed at room temperature or 4°C for 24 h, frozen and thawed three times, and stored at −70°C for 85 days. The stability evaluation was conducted for both LQC and HQC concentrations. The results in Table 3 indicate that the RSD and RE values for both compounds fall within an acceptable range, demonstrating the stability of PB1 and PB2 under these conditions. The stability of the processed samples was also tested, and the analyte was stable up to 24 h when it was stored at 4°C or room temperature.

Moreover, the stability of PB1 and PB2 in whole blood was determined using redundant TDM whole-blood samples. A clinical whole-blood sample was obtained and immediately divided into 10 aliquots and placed at room temperature (25°C) or 4°C. At the designated time point, one of the aliquots was removed and immediately centrifuged to obtain 100 μL of a plasma sample. Then, all plasma samples were stored at −70°C until further analysis. Two parallel samples were tested at each storage condition, and the results were expressed as the percentage ratios of PB1 or PB2 concentrations measured at different time points to the initial PB1 or PB2 concentration.

### 2.6 Clinical application of the HPLC–MS/MS method in a pharmacokinetic study

This study was approved by the Medical Ethics Committee of Shandong Provincial Hospital (No. 2021–356). Written informed consent was obtained from all patients or their legal representatives. Herein, we included 14 elderly patients (65≤ Age ≤95) who received intravenous PB injections (polymyxin B sulfate for injection, Shanghai First Biochemical Pharmaceutical Co., Ltd.) in the period between April 2021 and January 2023 for treating infections caused by multidrug-resistant Gram-negative bacteria. These patients were divided into two groups: in the first group, eight patients received 100 mg loading dose combined with 75 mg maintenance dose q 12 h, and in the other group, six subjects received a 150-mg loading dose and a 100-mg maintenance dose q 12 h. The duration of the intravenous treatment was 1 h. The dosage for all patients was decided by the clinician according to the clinical response. Patients undergoing plasma exchange, continuous renal replacement therapy, or extracorporeal membrane oxygenation were excluded. Clinical and demographic data (age, gender, body weight, pathogens, infection sites, and PB-treatment history) were collected from electronic medical records. Laboratory data, including serum creatinine (Scr), estimated glomerular filtration rate (eGFR), and creatinine clearance (CrCL), were collected on the sampling day. CrCL was calculated using the Cockcroft–Gault equation. On the third day after the administration of the loading dose, blood samples were collected at pre-dose (0 h) and 0.5, 1, 2, 3, 6, 8, and 12 h after the start of administration using EDTA tubes and processed to obtain plasma samples. A total of 112 blood samples were collected and detected, and all of these were stored at −70°C. After analysis by the validated methods, PK parameters were calculated by non-compartmental analysis using Phoenix WinNonlin software 8.3 (Certara, United States).

After completion of the entire sample analysis, the study data were validated by conducting an incurred sample reanalysis (ISR), as recommended by the guidelines referred in this study. A total of 13 samples were reanalyzed and quantified under freshly spiked calibration curve standards. The results were compared with the initial values. The 10% of the study samples for the first 1,000 samples needs to undergo ISR, and 2/3 of samples needs to be with ±20% difference.

## 3 Results

### 3.1 Method validation

#### 3.1.1 Specificity, selectivity, linearity, and LLOQ

**FIGURE 1:**
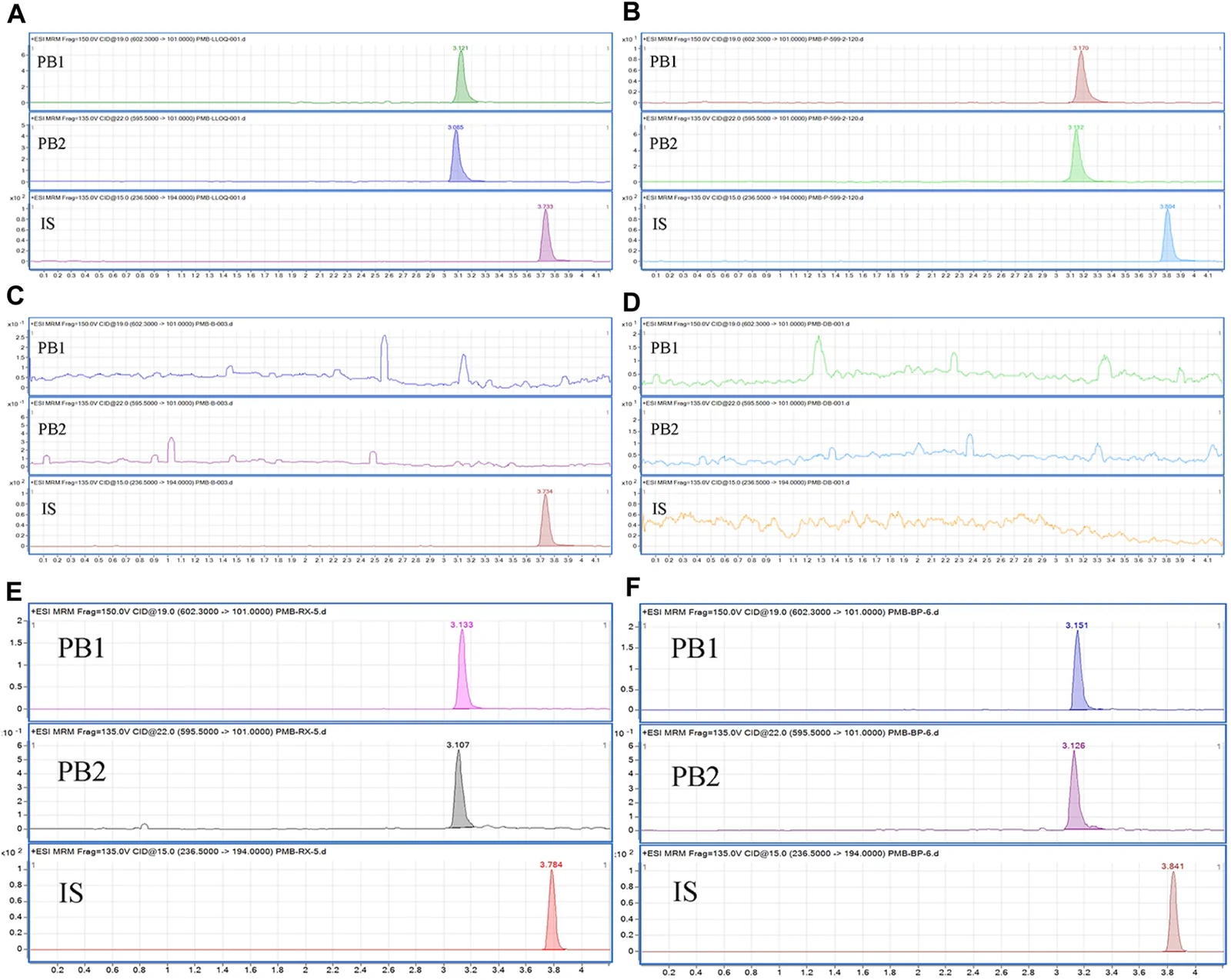
Typical chromatograms of **(A)** blank plasma samples spiked with PB1, PB2, and the IS at the LLOQ concentrations of the PBs; **(B)** IS-spiked plasma samples from patients receiving PB administration; **(C)** blank plasma samples spiked with the IS; **(D)** blank plasma; **(E)** blank hemolysed plasma samples spiked with analytes and IS; **(F)** blank plasma samples; blank lipemic plasma samples spiked with analytes and IS.

The specificity and selectivity of the method were investigated by analyzing blank plasma spiked with co-combined drugs and blank human plasma from six different sources to ensure that there were no interfering signals at the retention times of PB and IS. Figure 1 illustrates that no interferences from endogenous compounds were observed at the retention times of the analyte and IS.

The linear regression equation of the standard curve was as follows: *y* = 0.636210*x* + 0.008583 (*r* ^2^ = 0.99702043) for PB1 and *y* = 1.855529*x* + 0.027841 (*r* ^2^ = 0.9967636) for PB2 using 1/*x* ^2^ as the weight factor. Calibration curves constructed in three different experiments were used to assess the linearity, and the accuracies of the back-calculated concentrations of the calibration standards were within ±20% of the LLOQ concentration and ±15% of the other concentrations. The LLOQ values for PB1 and PB2 were 1.00 and 0.10 μg/mL, respectively (Figure 1).

#### 3.1.2 Accuracy and precision

The intra-day and inter-day accuracies for PB1 and PB2 were in the range of 93.29%–104.47% and 93.29%–102.35%, respectively, and the intra-day and inter-day RSD values were less than 10.46% and 13.40%, respectively. The results of accuracy and precision are shown in Table 1.

**TABLE 1:** Accuracy and precision of PB1 and PB2 in human plasma.

Analyte	Con. (μg/mL)	Intra-day				Inter-day			
		Mean (μg/mL)	Accuracy/%	RSD/%	RE/%	Mean (μg/mL)	Accuracy/%	RSD/%	RE/%
PB1	1.00	1.00 ± 0.01	100.29	9.65	+0.29	1.02 ± 0.07	102.35	6.86	+2.35
	2.00	1.87 ± 0.04	93.29	2.14	−6.71	1.94 ± 0.16	96.96	8.25	−3.04
	9.01	8.86 ± 0.21	98.47	2.32	−1.53	8.91 ± 0.55	98.96	6.20	−1.04
	18.02	18.80 ± 0.32	104.47	1.70	+4.47	18.37 ± 0.74	102.05	4.04	+2.05
PB2	0.10	0.10 ± 0.00	101.75	3.39	+1.75	0.01 ± 0.01	93.29	13.40	−6.71
	0.20	0.19 ± 0.02	96.54	10.46	−3.46	0.20 ± 0.02	98.51	9.88	−1.49
	0.92	0.87 ± 0.04	97.09	4.32	−2.91	0.91 ± 0.07	101.04	8.00	+1.04
	1.84	1.78 ± 0.04	99.03	2.13	−0.97	1.84 ± 0.09	102.19	4.82	+2.19

#### 3.1.3 Matrix effects and recovery

The IS-normalized matrix factors, which were calculated by dividing the matrix factors of PB1 or PB2 by that of the IS, were in the range of 85.36%–93.99%, with the RSD less than 10.24%; the recoveries of PB1 and PB2 ranged from 24.92% to 30.88%, with the RSD less than 5.21% (Table 2). The results of the matrix effect of PB1 and PB2 in lipemic and hemolysed plasma are shown in Table 3. The results suggest that the matrix-related interference of the established method was negligible, and the extraction efficiency is reproducible.

**TABLE 2:** Recoveries and matrix factors of PB1 and PB2 in human plasma (*n* = 6).

Analyte	Conc. (μg/mL)	Mean recovery		IS—normalized matrix effect	
		Mean ± SD	RSD/%	Mean ± SD	RSD/%
PB1	2.00	24.92 ± 1.09	4.37	92.78 ± 6.63	7.15
	9.01	28.10 ± 1.06	3.78	92.76 ± 2.90	3.13
	18.02	26.60 ± 0.39	1.48	85.97 ± 4.23	4.92
PB2	0.20	28.45 ± 1.48	5.21	93.99 ± 9.63	10.24
	0.92	30.88 ± 1.47	4.77	91.22 ± 3.36	3.68
	1.84	29.38 ± 0.93	3.18	85.36 ± 3.89	4.55

**TABLE 3:** Matrix effect of PB1 and PB2 in lipemic and hemolysed plasma (*n* = 6).

Analyte	Conc. (μg/mL)	IS—normalized matrix effect in lipemic plasma		IS—normalized matrix effect in hemolysed plasma		
		Mean ± SD	RSD/%	Mean ± SD		RSD/%
PB1	2.00	88.98 ± 3.99	4.48	82.80 ± 4.01		4.84
	9.01	79.39 ± 6.23	7.84	78.06 ± 5.39		6.90
	18.02	78.87 ± 2.82	3.58	79.67 ± 1.97		2.48
PB2	0.20	95.74 ± 8.52	8.90	98.88 ± 5.94		6.01
	0.92	85.84 ± 5.33	6.21	90.32 ± 11.38		12.60
	1.84	83.85 ± 2.76	3.29	85.76 ± 3.27		3.81

#### 3.1.4 Carryover and dilution integrity

No remarkable carryover was found in blank samples, following the upper LOQ (ULOQ) samples throughout the entire validation process. The mean PB1 concentration calculated from five replicates of the diluted PB1-containing plasma sample accounted for 97.24% of the theoretical PB1 concentration, with an RSD of 4.82%. Similarly, the mean PB2 concentration calculated from five replicates of the diluted PB2-containing plasma sample accounted for 99.76% of the theoretical PB2 concentration, with the RSD of 5.91%. These results indicate the credibility of the data even after a 2-fold dilution.

#### 3.1.5 Stability

**FIGURE 2:**
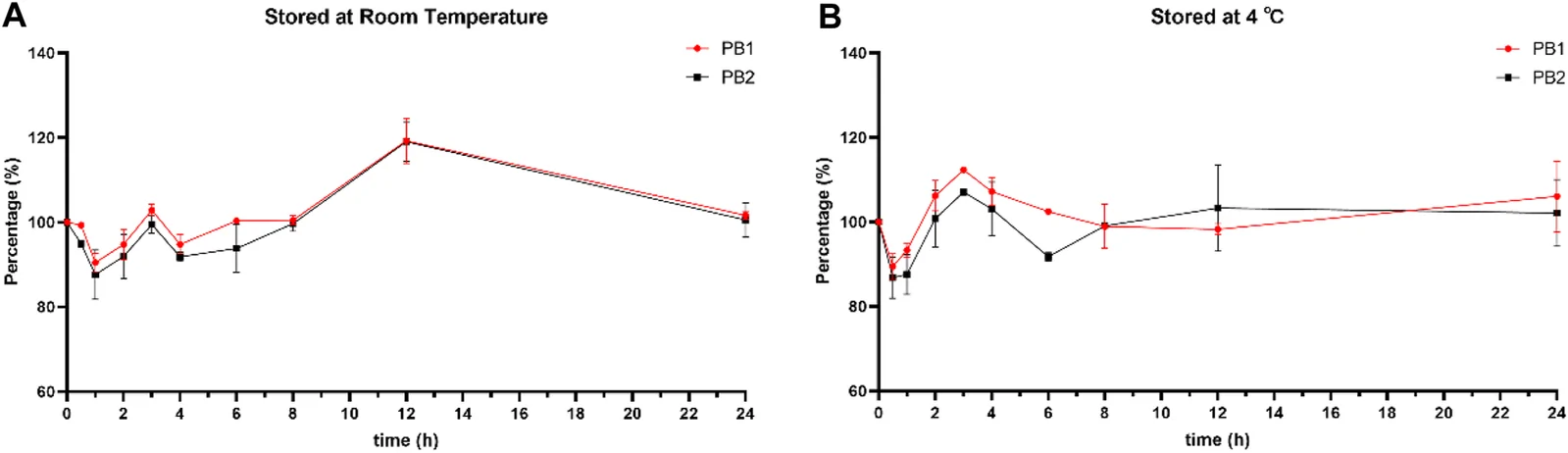
Stability of PB1 and PB2 in human blood stored at **(A)** 4°C or **(B)** room temperature (25°C).

The working solutions containing PB1 and PB2 at LQC and HQC concentrations remained stable after storage at −70°C for up to 7 days but not beyond 10 days. In addition, PB was stable in human plasma for 24 h at room temperature (25°C), for 24 h at 4°C (post preparative), after three freeze–thaw cycles, and for 85 days at −70°C. All the stability results are summarized in Table 4. The results of whole-blood samples are shown in Figure 2; Table 5.

**TABLE 4:** Stability of PB1 and PB2 in human plasma stored under different conditions (*n* = 3).

Storage condition	Analyte	Target concentration (μg/mL)	Accuracy and precision		
			Mean ± SD (%)	RSD (%)	RE (%)
Room temp. for 24 h	PB1	2.00	1.76 ± 0.22	12.20	−11.99
		9.01	9.48 ± 0.12	1.27	+5.18
		18.02	19.14 ± 2.05	10.72	+6.19
	PB2	0.20	0.19 ± 0.02	13.05	−4.90
		0.92	0.97 ± 0.04	3.65	+5.88
		1.84	1.88 ± 0.18	9.52	+2.51
4°C for 24 h	PB1	2.00	2.07 ± 0.06	2.72	+3.25
		9.01	8.35 ± 0.32	3.87	−7.38
		18.02	17.32 ± 0.21	1.21	−3.87
	PB2	0.20	0.22 ± 0.01	3.43	+6.37
		0.92	0.82 ± 0.03	3.34	−10.13
		1.84	1.75 ± 0.03	1.47	−4.68
Three freeze–thaw cycles	PB1	2.00	1.92 ± 0.19	10.03	−4.15
		9.01	9.30 ± 0.18	1.94	+3.09
		18.02	18.20 ± 0.59	3.26	+1.01
	PB2	0.20	0.20 ± 0.03	13.89	−4.41
		0.92	0.81 ± 0.03	3.32	−11.66
		1.84	1.68 ± 0.05	3.00	−8.28
−70°C for 85 days	PB1	2.00	1.95 ± 0.20	10.51	−2.50
		9.01	9.02 ± 0.24	2.61	+0.10
		18.02	18.37 ± 0.40	2.19	+1.95
	PB2	0.20	0.19 ± 0.02	8.84	−5.88
		0.92	0.80 ± 0.02	2.44	−12.53
		1.84	1.69 ± 0.05	3.09	−7.90

**TABLE 5:** Stability of PB1 and PB2 in human blood stored at 4°C or room temperature (25°C).

Time (h)	Stored at room temperature				Stored at 4°C			
	PB1 (μg/mL)		PB2 (μg/mL)		PB1 (μg/mL)		PB2 (μg/mL)	
0	6.89	6.66	0.66	0.6	4.69	4.72	0.42	0.41
0.5	6.83	6.63	0.62	0.56	4.29	4.12	0.38	0.34
1	6.13	6.13	0.54	0.55	4.42	4.35	0.38	0.34
2	6.7	6.15	0.62	0.53	5.1	4.89	0.44	0.39
3	7.16	6.78	0.65	0.58	5.25	5.32	0.45	0.43
4	6.42	6.42	0.59	0.55	5.13	4.95	0.45	0.4
5	6.89	6.71	0.58	0.58	4.82	4.82	0.39	0.37
8	6.97	6.65	0.65	0.59	4.46	4.85	0.4	0.42
12	7.95	8.19	0.75	0.73	4.56	4.68	0.4	0.45
24	7.05	6.72	0.67	0.58	4.69	5.28	0.41	0.44

### 3.2 Clinical application of the HPLC–MS/MS method in a PK study

**FIGURE 3:**
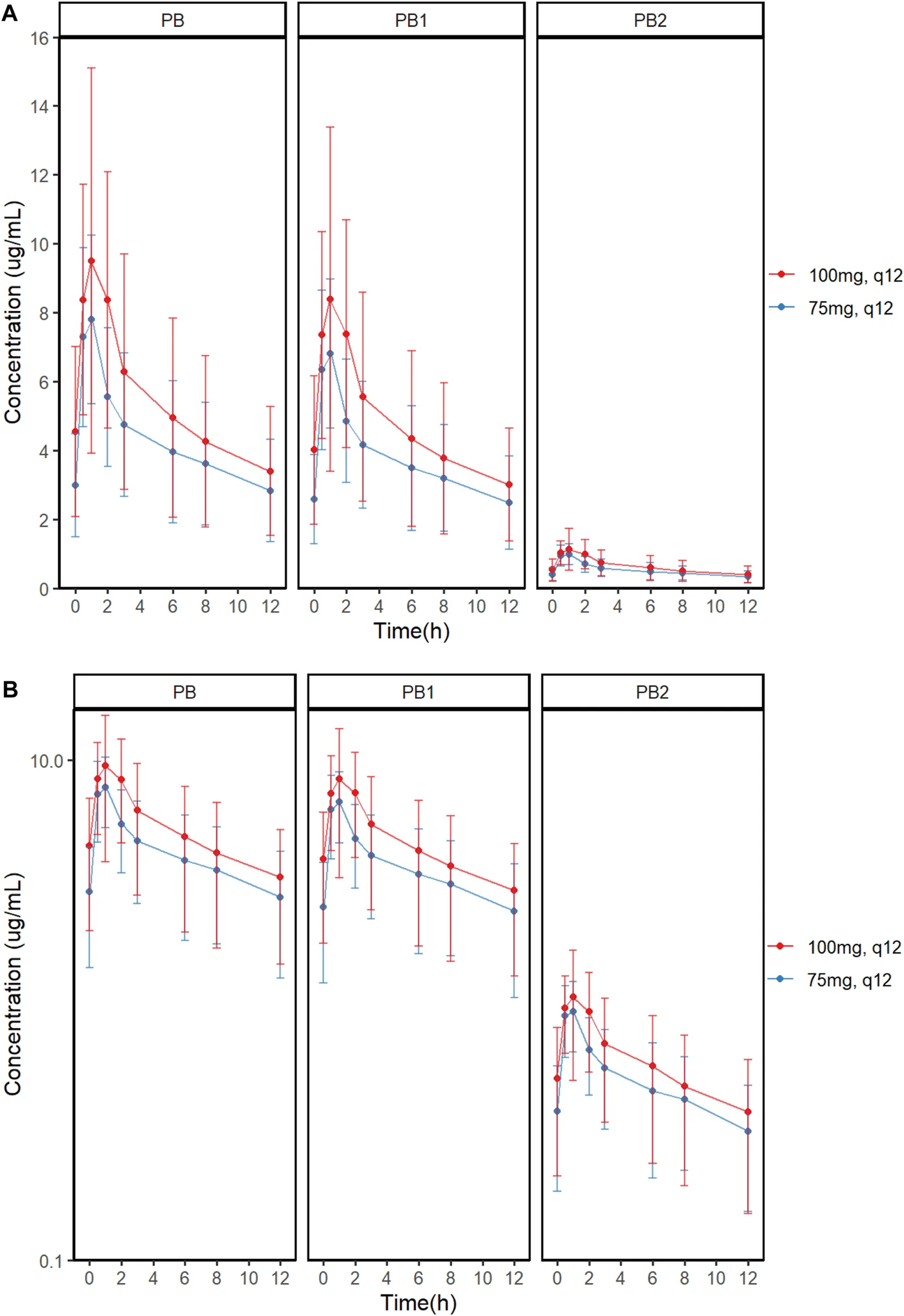
Linear **(A)** and semilog **(B)** mean plasma concentration–time curves for PB, PB1, and PB2.

After validation, the HPLC–MS/MS method was applied to the PK study of PB in elderly patients infected with multidrug-resistant Gram-negative bacteria. A total of 112 blood samples were collected from 14 elderly patients receiving intravenous PB treatment. Table 6 shows the demographic characteristics of the patients. Figure 3 shows the plasma concentration–time curves for the two groups of patients, and Table 7 presents the main PK parameters. The concentration of PB is the sum of the concentrations of PB1 and PB2 calculated, and it is based on the formula provided by Liu et al. (2023). The individual AUC_ss, 24 h_ values were estimated using the first-order elimination-based equation method (Begg et al., 1995; Pai et al., 2014; Liu et al., 2023). The mean AUC24 h_, ss_, C_av, ss_, and t_1/2_ of patients receiving a 75-mg maintenance dose were 117.70 μg h/mL, 4.14 μg/mL, and 11.01 h, respectively, and were 152.73 μg h/mL, 5.43 μg/mL, and 13.35 h for patients receiving a 100-mg maintenance dose.

**TABLE 6:** Demographic characteristics of elderly patients receiving PB treatment.

Characteristic	Dosage of polymyxin B	
	Maintenance dose 75 mg, q12 (n = 8)	Maintenance dose 100 mg, q12 (n = 6)
Age (yrs)	79.8 ± 8.2	72.8 ± 4.8
Gender		
Male	5	6
Female	3	0
Total body weight (Kg)	63.0 ± 10.4	67.5 ± 5.2
CrCL (mL/min)	95.5 ± 48.4	77.2 ± 49.9
Serum creatinine (μmol/L)	57.9 ± 25.6	111.8 ± 82.43
eGFR (mL/min)	89.4 ± 26.9	66.0 ± 39.1

Note: CrCL values were calculated with the Cockcroft–Gault formula; eGFR values were calculated with the CKD–EPI formula.

**TABLE 7:** PK parameters of PB (data were expressed as mean ± SD).

PK parameter	Dosage of polymyxin B	
	Maintenance dose 75 mg, q12 (n = 8)	Maintenance dose 100 mg, q12 (n = 6)
AUC_last_ (μg · h/mL)	48.07 ± 22.44	65.2 ± 34.25
AUC_ss, 24 h_ (μg · h/mL)	117.70 ± 37.03	152.73 ± 70.09
t_1/2_ (h)	11.01 ± 6.28	13.35 ± 5.79
C_max_ (μg/mL)	8.31 ± 2.35	10.65 ± 4.60
C_trough_ (μg/mL)	2.65 ± 1.32	3.40 ± 1.87
C_av, ss_ (μg/mL)	4.14 ± 1.74	5.43 ± 2.85
CL_ss_ (L/h)	2.10 ± 1.87	1.99 ± 1.12
V_ss_ (L)	22.76 ± 12.07	32.95 ± 19.51

Note: AUC_last_, area under the curve from the time of dosing to the time of the last measurable concentration; AUC_ss_, 24 h, area under the curve reflecting the steady-state plasma concentrations over a 24-h interval after dosing; t_1/2_, terminal half-life time; C_max_, peak plasma concentration; C_trough_, measurable concentration before the next dose is administered; C_av, ss_, average steady-state plasma concentration; CL_ss_, steady-state total body clearance; V_ss_, steady-state volume of distribution.

ISR was also performed after the completion of study sample analysis. A total of 13 samples (near Cmax or the elimination phase in the pharmacokinetic profile) were reanalyzed, and results are shown in Table 8. The concentration difference between the original and repeated values for the tested samples was less than 20%.

**TABLE 8:** Incurred sample re-analysis data on PB1 and PB2.

Sample	PB1			PB2		
	Initial concentration (μg/mL)	Re-assay concentration (μg/mL)	Difference (%)	Initial conc. (μg/mL)	Re-assay conc. (μg/mL)	Difference (%)
1	3.50	3.73	+6.50	0.48	0.47	−0.69
2	1.90	1.96	+3.34	0.29	0.29	−3.85
3	9.06	9.28	+2.35	1.33	1.30	−2.3
4	11.36	12.12	+6.72	1.64	1.56	−4.75
5	5.90	6.14	+4.15	0.81	0.76	−5.17
6	15.35	17.99	+17.19	1.88	1.98	5.12
7	2.00	1.90	−4.77	0.30	0.33	7.77
8	1.69	1.60	−5.33	0.25	0.25	−3.65
9	12.04	11.06	−8.17	1.65	1.43	−13.77
10	8.44	7.96	−5.71	1.11	1.18	5.53
11	2.72	2.46	−9.47	0.40	0.34	−14.49
12	3.81	3.58	−5.88	0.54	0.47	−12.72
13	1.55	1.61	+3.94	0.24	0.24	−0.46

## 4 Discussion

The commercial intravenous formulation of PB is a heterogeneous mixture wherein PB1, PB2, PB3, and PB1-ile are the primary bioactive constituents. According to studies conducted in animal models and healthy human volunteers, the four major components of PB have similar PK and pharmacodynamic (PD) characteristics (Manchandani et al., 2016; Liu et al., 2021). The studies of quantitation of PB are often based on the determination of PB1 and PB2 because these components account for >85% of the composition of PB (He et al., 2010). PB1 and PB1-ile share the same molecular weight, and they exhibit comparable response profiles and retention times (Liu et al., 2020) in the LC–MS/MS system. Therefore, in this study, the peaks corresponding to PB1 and PB1-ile were merged and reported as a single peak representing PB1.

A common challenge that should be taken into account when analyzing peptides is their nonspecific binding (NSB). Peptides tend to stick to various surfaces used during the whole analysis, such as pipette tips or vials, as well as to parts of the LC–MS system. Mostly, NSB is caused by either ionic or hydrophobic interactions. It typically results in non-linearity, poor sensitivity, carry-over, and peak tailing (Fage et al., 2019; Liu et al., 2020; Gaugain et al., 2021). NSB can be avoided by using organic solvents, incorporating structurally similar compounds or serum/plasma in the solvent, and employing suitable container materials such as polypropylene vials (John et al., 2004; Wilson et al., 2010). In the present study, water was used as the solvent for preparation of the stock solution and working solution, 0.1% FA was added to the above solvent, and a polypropylene tube was used as the container to effectively avoid NSB. A total of 0.1% FA was added in both phases A and B to prevent NSB in the LC–MS/MS system.

The use of stable isotope-label IS or structural analogs is an ideal choice for LC−MS/MS bioanalysis, especially in the case where matrix effects cannot be eliminated. However, isotope-label IS of PB is unavailable. Colistin was initially considered the IS candidate for this experiment. However, it showed a problem of stability in our method when stored in the autosampler. It has been proven that both colistin A and colistin B cannot be stable when stored at 25°C for 6 h (Matar and Al-Refai, 2020). Ultimately, carbamazepine, which has a stable extraction recovery, chromatography behavior, and mass spectrometry response, was selected as IS (Mao et al., 2017). The retention time of carbamazepine was close to those of PB1 and PB2 under the method established in this study.

The retention times of PB1, PB2, and IS are in the re-equilibration of gradient program, which may cause a shift in retention time. The mean retention times of PB1, PB2, and IS in all precision and accuracy runs are 3.123 min, 3.087 min, and 3.744 min for batch 1; 3.157 min, 3.121 min, and 3.788 min for batch 2; and 3.137 min, 3.100 min, and 3.755 min for batch 3; with 1.44%, 1.43%, and 1.90% for PB1, PB2, and IS, respectively. The variation in the mean retention time intra batch is mainly because of the preparation difference of the liquid phase between the batches. Additionally, the whole-blood samples were stable when stored for 24 h at both 4°C and room temperature, which support the TDM samples being transferred from clinics to laboratories and stored overnight.

Compared to the administration of a fixed dose of PB, the administration of a body-weight-based dose of PB increases the efficacy of the antibiotic (Sandri et al., 2013; Miglis et al., 2018; Tsuji et al., 2019; Wang et al., 2021a). However, clinicians usually administer a fixed dose of PB due to challenges in obtaining accurate body weight information about critically ill patients. Moreover, fixed loading and maintenance doses of polymyxin B appear more appropriate than weight-based dosing regimens in patients within the 45–90-kg BW range (Hanafin et al., 2023). This study is the first to report the PB pharmacokinetics parameters in critically ill elderly patients receiving fixed intravenous PB doses. Herein, C_av, ss_ of elderly patients was higher than the previously reported values for critically ill adult patients, and CL_ss_ values in adult patients are higher than those of the elderly patients (Wang et al., 2020a). The above study did not show the renal function indicators. Generally, in the elderly, glomerular function declines with age, and renal physiological functioning continues to deteriorate with increasing age (Olson, 2003). This may cause the higher exposure of PB in the elderly. Recent population PK studies indicated a typical value of CL of 1.87 L/h in elderly patients (Wang et al., 2022), which is slightly lower than that of adult patients (ranging between 1.9 and 2.5 L/h) (Manchandani et al., 2018; Wang et al., 2020b; Yu et al., 2021). In the above population PK study, a fixed maintenance dosage of 50 mg and 75 mg for the elderly may maximize efficacy while showing a lower probability of AUC_ss,24h_ greater than 100 μg h/mL, which is lower than that recommended by the international guideline for adults (Tsuji et al., 2019). Therefore, a lower dose might be more appropriate for the elderly.

The recommended AUC_ss, 24 h_ range of PB, which is based on the therapeutic window of the drug, is between 50 and 100 μg h/mL (Tsuji et al., 2019; Liu et al., 2023). The average AUC_ss, 24 h_ values for two groups (117.70 μg h/mL and 152.73 μg h/mL) were both higher than that range. In case of the two groups, patients had AUC_ss, 24 h_ ranged from 67.05 to 177.64 μg h/mL and 74.42 to 233.79 μg h/mL for 75 mg q12 and 100 mg q12, respectively. AUC_ss, 24 h_ values exceeding 100 μg h/mL were a good predictor for an increasing risk of acute kidney injury and nephrotoxicity (Lakota et al., 2018; Maniara et al., 2020; Wang et al., 2021b). This study simply recorded kidney function indicators in the two groups during therapy; after an average of 5.29 days of therapy, the mean Scr value of patients receiving a 75-mg maintenance dose increased from 50.8 to 57.9 μmol/L, and the mean eGFR value of the patients decreased from 93.83 to 89.40 mL/min. In patients who received a 100-mg maintenance dose, the mean Scr value increased from 74.30 to 111.82 μmol/L, and the mean eGFR value of the patients decreased from 83.80 to 66.00 mL/min after an average of 3.83 days. Patients receiving a 100-mg maintenance dose of PB have a higher risk of nephrotoxicity. It is worth noting that the development of renal failure during therapy directly influences clinical outcome and prognosis, and the degree of AKI developed is directly proportional to the increase in the risk of death (Gomes et al., 2018). These results indicated that both the dosages are too high for the elderly, and the analysis of the PB exposure–effective/nephrotoxicity relationship in the elderly should be more comprehensive. There are some limitations in this work. First, the whole time for each injection is 7.5 min, which is quite long for such a HPLC–MS/MS method compared with existing methods. Furthermore, crosstalk of PB1 and PB2 should be considered due to the high structural similarity. Structural analogs had a response at the same MRM channels, resulting in a high risk of detection crosstalk, and PB1 and PB2 were susceptible to crosstalk at ion channels in m/z 602.3 → 101.0 for PB1 and m/z 595.5 → 101.0 for PB2. To avoid the MS/MS crosstalk among the above-mentioned analytes undermining their detection sensitivity and quantitative accuracy, each analyte should be quantified with satisfactory chromatographic separation. Hee et al. (2017) separated four major components of PB using a specific column, and no crosstalk signal was observed in LLOQ chromatography. However, crosstalk still should be measured in a high concentration level. Further study of the PB method should investigate the crosstalk of PB components. For PK application, no female patients were enrolled in the high-dosage group. However, studies have indicated that gender has no significant effect as a covariate on the PK parameters of polymyxin B (Wang et al., 2020b; Hanafin et al., 2023). In addition, the relationship between the dose and safety was discussed through exposure and therapeutic window; however, these two regimens are not adequately evaluated for efficiency.

Considering the narrow therapeutic window and serious nephrotoxicity of PB, TDM should be considered because of high variability of PK, and renal function should be monitored for patients during the treatment. The method established in this study can be used to monitor PB plasma concentrations. Population PK model and empirical Bayes estimation were beneficial for guiding individualization of dosing to maximize PTA while minimizing toxicity and improving the benefit/risk ratio during dosing.

## 5 Conclusion

A simple HPLC–MS/MS method with a simple sample-pretreatment method based on protein precipitation was developed to determine PB concentrations in human plasma. This method was validated according to the guidelines from the Chinese Pharmacopeia and ICH M10 for the following parameters: specificity, selectivity, linearity, accuracy, precision, matrix effects, recovery, carryover, dilution integrity, and stability. Subsequently, it was successfully applied for studying PB pharmacokinetics in elderly patients infected with multidrug-resistant Gram-negative bacteria.

## Data Availability

The original contributions presented in the study are included in the article/supplementary material; further inquiries can be directed to the corresponding authors.
